# Validation and reliability analysis of the Questionnaire on the Emotional Impact of Vertigo-CIEV version translated to the Brazilian Portuguese language

**DOI:** 10.1590/2317-1782/20232022176en

**Published:** 2023-12-04

**Authors:** Maria Luiza di Carlo Riato, Vanessa Brito Campoy Rocha, Raquel Mezzalira, Guita Stoler, Maria Isabel Ramos do Amaral

**Affiliations:** 1 Programa de Graduação em Fonoaudiologia, Faculdade de Ciências Médicas, Universidade Estadual de Campinas - UNICAMP - Campinas (SP), Brasil.; 2 Departamento de Desenvolvimento Humano e Reabilitação, Faculdade de Ciências Médicas, Universidade Estadual de Campinas - UNICAMP - Campinas (SP), Brasil.; 3 Departamento de Otorrinolaringologia, Cabeça e Pescoço, Faculdade de Ciências Médicas, Universidade Estadual de Campinas - UNICAMP - Campinas (SP), Brasil.

**Keywords:** Dizziness, Vertigo, Symptom Assessment, Emotional Distress, Questionnaires

## Abstract

**Purpose:**

To determine the internal consistency and reliability of the “Questionário de Impacto Emocional da Vertigem (CIEV)” and to validate the instrument with respect to the Dizziness Handicap Inventory (DHI) in a sample of individuals with balance disorders.

**Methods:**

38 subjects participated in the study, males and females, aged from 23 to 85 years, who presented dizziness, vertigo, and/or falls complaints and attended to the Vestibular Disorders clinic at the University Hospital. Individuals with hearing complaints and/or tinnitus unrelated to dizziness, previous psychiatric comorbidities, and/or cognitive impairments were excluded. We performed an anamnesis and collected complementary data from the medical records. After that, the self-perception questionnaires, DHI, and CIEV, were applied. Statistical analysis was performed in which the Cronbach’s alpha verified the internal consistency of the CIEV. Reliability and validity of the CIEV related to the DHI were calculated using Intraclass Correlation Index (ICC) and Pearson’s correlation test, respectively.

**Results:**

There was a statistically significant correlation between the scores obtained, for both reliability and validation analysis (p<0.001). The mean ICC showed a moderate correlation between the total scores (0.695) and a strong correlation with the physical, emotional, and functional DHI domains (0.706 to 0.869), being the emotional aspect the highest degree (0.869). Pearson’s correlation showed strong correlation between the total scores (r=0.820) and varied from moderate to strong, with strongest correlations to the DHI emotional domain (r=0.788).

**Conclusion:**

The outcomes illustrate important contribution to validation parameters to consider clinical use of the CIEV in the Brazilian population, aiming to identify emotional aspects in patients with balance disorders.

## INTRODUCTION

Vertigo and dizziness occur when there is a disruption in the integration of sensory information from the visual, vestibular, and proprioceptive systems^(1,2)^. This condition is marked by changes in how sensory nerves process information, resulting in feelings of instability and disorientation. These symptoms can be attributed to various underlying causes and can have a significant impact on daily activities and overall quality of life^(2,3)^

In recent years, the field of neuro-otology, or vestibular sciences, which studies balance disorders, has presented significant clinical and scientific advancements. Current consensus and clinical guidelines provide important information for an appropriate diagnosis of the different conditions that affect the vestibular system. These guidelines include physical and instrumented assessments, along with advanced imaging tests to enhance accuracy. However, despite these advancements, the patient's clinical history and self-reported experiences remain essential for an accurate diagnosis and effective planning and monitoring of therapeutic interventions for individuals with vestibular disorders ^(4)^.

Among the various factors that may be associated with dizziness, the emotional aspects are highlighted in this study. In recent decades, studies have analyzed the relationship between psychological issues and vertigo^(5,6)^. The existence of a cause and effect relationship or concomitance of symptoms has been investigated due to the common pathways and connections between the vestibular system and the limbic system^(5)^. Studies indicate that individuals with otoneurological complaints are more likely to have psychological problems and a more stressful lifestyle than patients without these complaints and individuals without complaints possibly have better physiological mechanisms to deal with stress^(7)^.

Understanding the emotional and otoneurological symptoms and the impact of one symptom on the other may ensure a better management of the clinical case^(5)^. It is not an easy task and, many times, the assessment of the symptoms may not be enough. Even in asymptomatic periods, dizziness can compromise different aspects of the daily life due to anxiety and fear of anticipating a next episode, more than the occurrence of the symptom itself^(8)^. As a result, the diagnostic process may have limitations in detecting psychological issues linked to dizziness, making it challenging to identify this connection during the initial evaluation of vertigo patients, as anxiety might or might not be apparent at that stage^(9)^.

Self-perception questionnaires have been recommended as instruments to quantify and analyze subjective symptoms that are hard to characterize, such as dizziness and its different impacts on the quality of life^(10)^. The Dizziness Handicap Inventory (DHI) questionnaire, proposed by Jacobson and Newman^(11)^, is the most common instrument in research and clinical practice for this purpose. It has been validated in different countries, including Brazil^(12)^ and is still widely studied. Recently, a reduced version has been proposed by the authors to facilitate an assessment of such aspects during clinical practice^(13)^.

As a proposed clinical instrument of this nature, the “Questionnaire to Assess the Emotional Impact of Vertigo - CIEV” was developed and validated in the Spanish language^(14)^ to measure the risk for pathological anxiety conditions secondary to vertigo based on the self-perception of symptoms as a complementary strategy to diagnostic assessment. In Brazil, the CIEV had its original version freely translated by the authors and published in Portuguese^(15)^. However, so far, no validation study has been conducted in Brazil for this potential instrument, whose clinometric properties and construct validity must be studied and discussed.

Our study hence aimed to determine the internal consistency and reliability of the “*Questionário de Impacto Emocional da Vertigem* (*CIEV*)*”* and validate the instrument in relation to the Dizziness Handicap Inventory (DHI), in a sample of patients with vestibular disorders without prior diagnosis of psychiatric alterations treated at a university hospital.

## METHODS

### Study type and site, ethical aspects

This is a quantitative, descriptive and cross-sectional reliability and validity study. Data collection was conducted at the otoneurology clinic of the institution’s university hospital after approval by the Research Ethics Committee (#2,344,836). All individuals agreed to participate as volunteers and signed an informed consent form (ICF).

### Study participants and inclusion criteria

The participants were selected from the outpatient clinic routine and evaluated on the day of the first visit. The main experimenter was present during the visits with the responsible medical team and, at the end of the consultation, the patients who met the inclusion criteria were invited to participate.

The inclusion criteria were: male and female individuals aged over 18 years old and who had complaints related to dizziness/vertigo and a previously self-known history of imbalance and/or fall. Participants with any degree of cognitive impairment that impeded their ability to comprehend and respond to the questionnaires were excluded from the study, as well as individuals who presented exclusively a hearing complaint and/or tinnitus, without dizziness. Individuals reporting prior psychiatric comorbidities in the anamnesis and/or medical records were also excluded.

### Sample characterization

Data for sample characterization were obtained from the following sources:

- Otoneurological anamnesis: aspects associated with the complaint (type of dizziness and complaint time), history of previous falls in the last 12 months before the assessment day, associated auditory symptoms (hearing loss and/or tinnitus), medications used, and general health.
- Medical records: data from the anamnesis were confirmed, hypotheses for the vestibular lesion topography were attributed to each case, and the presence of comorbidities was analyzed. When necessary, discussions were held with the medical team to confirm the information.

The type of dizziness was classified according to the International Classification of Vestibular Disorders^(16)^, as follows: non-spinning dizziness, vertigo, postural symptoms, and vestibulovisual symptoms. Data from the hypothesis for every participant’s vestibular lesion topography, collected from the medical records after the medical evaluation, were grouped into peripheral, central, mixed, cervical or to-be-clarified lesion topography. The mixed category included diseases of vascular, metabolic, hormonal causes and combined peripheral and central causes. In addition, to-be-clarified lesion topography included proprioceptive etiologies, inconclusive and/or normal otoneurological exams.

The sample consisted of 38 individuals aged 23 to 85 years, mean age of 58.66 years (±16.37), 25 (66.79%) were female patients. Complaint time ranged from 1 week to 4.87 years. Considering the last 12 months before patient assessment, 16 (38%) participants reported prior dizziness-related fall, 23 (60.53%) had some type of hearing loss and 26 (68.42%) reported tinnitus. Table 1 shows sample characterization in terms of type of dizziness reported, comorbidities, and distribution of the hypothesis for every participant’s vestibular lesion topography. Some subjects reported more than one type of complaint and more than one comorbidity. In addition, the cervical hypothesis was identified as a single topographical hypothesis in only two participants, and six patients presented more than one hypothesis associated with cervical lesion topography.

**Table 1:** Absolute distribution and relative frequency of the classification based on the type of complaint, topography of the vestibular lesion, and comorbidities identified in each case, considering data obtained from medical records

**Type of dizziness**	**n**	**%**	**Topographic distribution**	**n**	**%**	**Comorbidities**	**n**	**%**
Vertigo	24	48.97	Mixed	22	50.00	Endocrine	16	25.80
Postural symptoms	17	34.70	Cervical	8	18.18	Cardiovascular	15	24.20
Non-spinning dizziness	7	14.28	Central	7	15.90	Rheumatic	8	12.90
Vestibulovisual symptom	1	2.04	To be clarified	4	9.10	Neurological	7	11.30
-	-	-	Peripheral	3	6.81	Hematological	5	8.06
-	-	-	-	-	-	Respiratory	4	6.45
-	-	-	-	-	-	Autoimmune	3	4.84
-	-	-	-	-	-	Obesity	2	3.23
-	-	-	-	-	-	Lymphatic	1	1.61
-	-	-	-	-	-	Cancer	1	1.61
**Total**	**49**	**100.0**	**Total**	**44**	**100.0**	**Total**	**62**	**100.0**

Caption: n = absolute frequency

### Data collection

Prior to data collection, a pilot study was conducted to train and standardize the application of study questionnaires. First, the researcher explained the questionnaire to the participant and read the questions together with the participant. After the answer, the researcher marked the chosen option on the protocol sheet. The two questionnaires applied are described below:

- Dizziness Handicap Inventory (DHI)^(12)^: consisting of 25 questions, which assess three domains related to the possible loss/impact on the quality of life caused by dizziness. There are seven questions addressing physical aspects, nine questions focusing on emotional aspects, and another nine questions related to functional aspects. Possible answers are “always” (4 points), “sometimes” (2 points), and “no” (zero points). The final score is the sum of all points, ranging from 0 to 100. In addition, scores by physical, functional, and emotional domains were also calculated. Classification regarding the level of impact caused by dizziness is based on the total score and divided by degrees of handicap; results from 0 to 30 points indicate low emotional impact, 31 to 60 points indicate medium impact, and 61 to 100 points indicate high impact.
- “*Questionário de Impacto Emocional da Vertigem* (*CIEV*)”^(15)^: consisting of 19 questions divided into three parts: Part I is introductory regarding the definition of dizziness and vertigo reported by the patient and the question is not scored. The other 18 questions are distributed in Part II - Experience during episodes of vertigo/dizziness and Part III - Situations experienced regarding the associated degree of distress. In Parts II and III, the three response options have a score of 0, 1 or 2, with 0 indicating “Never” or “No, that never happened to me,” 1 attributed to “Sometimes” or “Yes, that makes me a little distressful,” and 2 attributed to “Often” and “Yes, that makes me very distressful.” Then, the final score ranges from 0 to 36 points. Results above 16 points are classified as risk for pathological anxiety and/or risk for a worse prognosis in the treatment of dizziness^(14,15)^. Chart 1 shows the full CIEV questionnaire.

**Chart 1:** Questionnaire to Assess the Emotional Impact of Vertigo - CIEV

**Questionário de impacto emocional da vertigem - CIEV**

Dados pessoais:
Nome completo: ______________________________________ DN: __________ idade: ________
Profissional responsável: __________________________________________________________

**I) Introdução:**
Entendemos por vertigem como a sensação de rotação do próprio corpo ou do entorno geralmente é um movimento rotatório). Exemplo: “As coisas giram...” Por outro lado, a tontura refere-se a uma sensação de desequilíbrio. Exemplo: “Andar nas nuvens...”. Qual dessas duas definições coincide mais com o que você sente?
() Vertigem () Tontura

**I) Responda de acordo com a sua experiência durante os episódios de vertigem/tontura:**

1. Enquanto estava com vertigem/tontura sentiu que estava perdendo controle sobre seu corpo?
NUNCA () AS VEZES () MUITAS VEZES ()

2. Enquanto estava com vertigem/tontura pensou que poderia desmaiar ou desfalecer?
NUNCA () AS VEZES () MUITAS VEZES ()

3. Enquanto estava com vertigem/tontura se sentiu desprotegido, sem ninguém para te socorrer? |
NUNCA () AS VEZES () MUITAS VEZES ()

4. Enquanto estava com vertigem/tontura sentiu ansiedade e medo de ser “dominado”?
NUNCA () AS VEZES () MUITAS VEZES ()

5. Enquanto estava com vertigem/tontura teve sintomas como taquicardia, sudorese ou asfixia?
NUNCA () AS VEZES () MUITAS VEZES ()

6. Enquanto estava com vertigem/tontura e ao sentir que havia muitas pessoas no local, seu mal-estar aumentava?
NUNCA () AS VEZES () MUITAS VEZES ()

7. Enquanto estava com vertigem/tontura sentiu que estava melhor na sua casa do que fora dela?
NUNCA () AS VEZES () MUITAS VEZES ()

8. Enquanto estava com vertigem/tontura sentia-se melhor se alguém de confiança estivesse
perto de você?
NUNCA () AS VEZES () MUITAS VEZES ()

|-----------------------------------------------------------------------------------------------|
Com a presença da sintomatologia de vertigem/tontura, muitas pessoas experimentam mudanças na forma de ser ou agir que as fazem sentir-se realmente “diferentes” de como sempre foram. A seguir é detalhada uma série de experiências frequentes de pacientes com problemas semelhantes aos seus.

**II) Responda se isso já aconteceu com você e, se a resposta for positiva, o quanto você se sente angustiado ao perceber essa mudança na sua maneira de ser.**

**1. Sente estar dependendo mais da ajuda ou da companhia de outras pessoas?**
A) Não, isso nunca me aconteceu. ()
B) Sim, e isso me deixa um pouco angustiado. ()
C) Sim, e isso me angustia muito. ()

**2. Sente que está mais sensível? (Ex.: Chora com mais facilidade,...)**
A) Não, isso nunca me aconteceu. ()
B) Sim, e isso me deixa um pouco angustiado. ()
C) Sim, e isso me angustia muito. ()

**3. Sente estar mais irritado, menos tolerante com as pessoas?**
A) Não, isso nunca me aconteceu. ()
B) Sim, e isso me deixa um pouco angustiado. ()
C) Sim, e isso me angustia muito. ()

**4. Sente que está mais medroso, que se assusta com mais facilidade do que antes?**
A) Não, isso nunca me aconteceu. ()
B) Sim, e isso me deixa um pouco angustiado. ()
C) Sim, e isso me angustia muito. ()

**5. Você pensa na possibilidade de sofrer doenças graves?**
A) Não, isso nunca me aconteceu. ()
B) Sim, e isso me deixa um pouco angustiado. ()
C) Sim, e isso me angustia muito. ()

**6. Sente temor de ficar sozinho em casa ou viajar sem companhia?**
A) Não, isso nunca me aconteceu. ()
B) Sim, e isso me deixa um pouco angustiado. ()
C) Sim, e isso me angustia muito. ()

**7. Você costuma evitar os lugares cheios de gente? (supermercados, cinemas, shoppings, etc)**
A) Não, isso nunca me aconteceu. ()
B) Sim, e isso me deixa um pouco angustiado. ()
C) Sim, e isso me angustia muito. ()

**8. Você acha que está suportando menos as “pressões” profissionais e familiares?**
A) Não, isso nunca me aconteceu. ()
B) Sim, e isso me deixa um pouco angustiado. ()
C) Sim, e isso me angustia muito. ()

**9. Você sente que fica difícil “lidar” com situações simples, que antes você controlava melhor?**
A) Não, isso nunca me aconteceu. ()
B) Sim, e isso me deixa um pouco angustiado. ()
C) Sim, e isso me angustia muito. ()

**10. Você se sente menos forte, com menos “capacidade para lutar” do que antes?**
A) Não, isso nunca me aconteceu. ()
B) Sim, e isso me deixa um pouco angustiado. ()
C) Sim, e isso me angustia muito. ()

**Escore: _________**

“Nunca” e “Não, isso nunca me aconteceu” = zero pontos
“Às vezes” e “Sim, e isso me deixa um pouco angustiado” = um ponto
“Muitas vezes” e “Sim, e isso me angustia muito” = dois pontos
|-----------------------------------------------------------------------------------------------|

### Statistical analysis

Data were analyzed through descriptive and inferential statistics, using SPSS 25.0, Minitab 16, and Excel Office 2010. Normality of quantitative variables for the main outcome was confirmed using the Shapiro-Wilk test and 5% significance level for all inferential analyses. Statistically significant values (p≤0.05) were highlighted in bold. 

For the description of quantitative variables, measures of central tendency (mean), measures of variability (standard deviation), and measures of position (minimum and maximum) were calculated. For the description of nominal qualitative variables, absolute frequency and relative frequency were calculated.

Cronbach’s alpha coefficient was calculated to analyze the internal consistency of the CIEV questionnaire data, based on the measure of mean correlation between the questions. The maximum value of this coefficient is 1.0 and the higher the value, the stronger the internal consistency of data. Alpha values above 0.7 are considered acceptable. This analysis considered the total CIEV score and the removal of every question.

The intraclass correlation coefficient (ICC) was calculated to assess the reliability of the study protocol and the validity was assessed using the Pearson correlation test, both analyses by comparing the total score of CIEV to the total score, and by domain as obtained from the DHI protocol. ICC and correlation values range from 0 to 1, with values closer to 1 indicating a better degree of correlation. In ICC calculation, in addition to the mean value, the ICC based on the lower and upper limits of the adopted confidence interval was also presented.

For the correlation coefficients (r) and ICC the following ranges were considered: |0.10| to |0.39| - weak correlation, |0.40| to |0.69| - moderate correlation, |0.7| to |1.00| - strong correlation^(17)^.

## RESULTS

Table 2 shows the sample performance in the CIEV, considering the final score and risk classification for pathological anxiety. Table 3 shows the sample performance in the DHI questionnaire, considering the total score, score in each domain, and classification of dizziness impact degree (handicap).

**Table 2:** Description of the score obtained in the CIEV and the risk classification for pathological anxiety (n=38)

Questionnaire to Assess the Emotional Impact of Vertigo (CIEV)						
Score	n	Min	Max	Mean	Median	Standard deviation
	38	5.0	35.0	20.39	20.00	8.48
**Classification**	**N**			**%**		
**Not a risk**	8			21.05%		
**Risk**	30			78.95%		

Descriptive analysis

Caption: n = absolute frequency; % = percent relative frequency; Min = minimum; Max = maximum

**Table 3:** Description of the score obtained in the DHI questionnaire and the classification of the impact of perceived handicap, considering the total classification and the functional, physical and emotional domains (n=38)

	**Score**							
									**Min**		**Max**		**Mean**		**Standard deviation**	
**Functional**	0		36		17.95		9.05	
**Physical**	0		28		16.90		7.81	
**Emotional**	0		36		14.62		10.60	
**Total**	16		92		51.47		23.00	
	**Low**		**Moderate**		**High**		**Total**	
**Classification (handicap)**	**n**	**%**	**n**	**%**	**n**	**%**	**n**	**%**
	9	23.68	15	39.47	14	36.84	38	100

Descriptive analysis

Caption: n = absolute frequency; % = percent relative frequency; Min = minimum; Max = maximum

**Figure 1:**
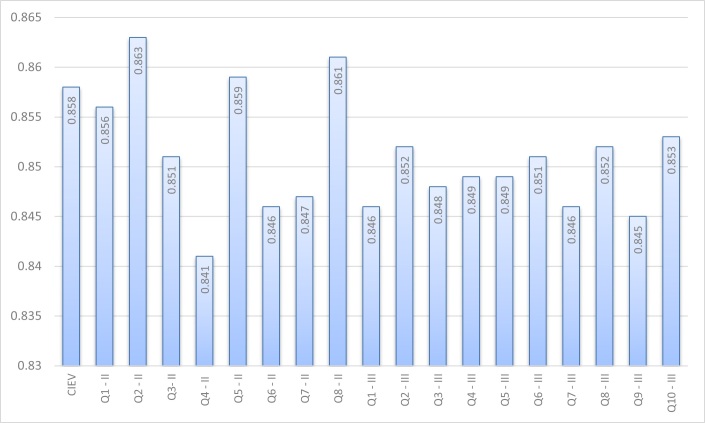
Internal consistency analysis using Cronbach’s alpha for the CIEV protocol (n = 38)

Figure 1 shows the internal consistency analysis of the CIEV questionnaire. The high alpha value obtained for the total score (0.858) remained stable, ranging from 0.841 to 0.893 based on the analysis with the removal of every question.

Table 4 shows the reliability and validity data of the CIEV protocol in relation to the total score and by DHI domain. The findings were statistically significant (p<0.001), and the mean values related to the total score showed a moderate correlation for instrument reliability (0.695) and strong correlation for instrument validity, also based on the total score (0.820).

**Table 4:** Reliability and validation analysis of the CIEV protocol in relation to the total score and domains assessed by the DHI protocol

**DHI**	**Reliability - ICC**				**Validation**	
	**ICC**	**p value**	**Lower limit**	**Upper limit**	**R**	**p value**
**Total score**	0.695	**<0.001**	0.413	0.841	0.820	**<0.001**
**Domains**						
**Physical**	0.706	**<0.001**	0.434	0.847	0.547	**<0.001**
**Emotional**	0.869	**<0.001**	0.749	0.932	0.788	**<0.001**
**Functional**	0.809	**<0.001**	0.632	0.901	0.681	**<0.001**

Caption: ICC = intraclass correlation index; Lim = Limit; R = correlation coefficient/Pearson correlation test

## DISCUSSION

Self-report questionnaires, especially those assessing skills or domains leading to a final score, such as the CIEV, are considered clinically valuable because they possess well-established clinometric properties. These properties, including reliability, validity, and accuracy data, contribute to their credibility and usefulness in clinical settings^(18)^. In this study, the CIEV analysis was based on the study of its construct reliability and validity, using internal consistency measures and degrees of correlation with the DHI, a questionnaire developed from a similar theory.

The DHI was selected because it is a validated and consolidated instrument for clinical use. Studies have demonstrated it is a reliable, comprehensive, easy-to-apply questionnaire suitable for the clinical evaluation of patients with dizziness, measuring the degree of impact on the quality of life that may be specifically related to vestibular disorders^(12,19)^. Even though it has been widely used and studied, the literature still highlights gaps and aspects for further studies from the DHI and other similar questionnaires, which justifies the development and validation of new instruments. Few studies have explored the correlation between scores and specific vestibular disorders or the clinical status of vestibular lesions^(20)^.

Regarding the results of our study given the performance of participants in each applied questionnaire, 30 individuals (78.95%) were at risk of developing pathological anxiety in the CIEV scores and 29 (76.31%) had altered scores in the DHI. Of all 30 individuals with altered scores in the CIEV, 25 (83.3%) were also classified as medium and high impact in the DHI. The sample performance agrees with results of studies that found an association between emotional aspects and dizziness^(5-9)^, particularly anxiety^(21)^.

The described performance is also consistent with the original study for the CIEV development and validation^(14)^. The authors assessed the emotional aspects of patients with persistent dizziness even after treatment using the CIEV. Of all 183 individuals assessed, 157 (86%) had a confirmed history of pathological anxiety and, of these, 60 (69.7%) had altered results in the CIEV. Despite the methodological differences in the sample characteristics in relation to the sample of our study, the investigation demonstrated an association between the symptoms and highlighted the fact that validation studies were required for the complementary use of CIEV in clinical practice.

Internal consistency is an indicator of measurement reliability and instrument stability, demonstrated through the mean correlation analysis between the answers of participants^(22)^. Data showed high internal consistency between the protocol questions and the total score. When assessing data with removal of every and each question, stability was confirmed with alpha values remaining high. The lowest value was obtained in question 4 related to the feeling of fear after the onset of dizziness, but still a high value (0.841).

In the ICC analysis, statistically significant values (p<0.001) were found in relation to the total DHI score (r=0.695) and among the three dimensions assessed (physical, functional, and emotional dimensions), with special attention to the emotional domain, which presented the highest mean degree of strong positive correlation (r=0.869). When considering the variation of lower and upper limit values based on a 95% confidence interval, the emotional domain showed a lower variation, demonstrating a strong correlation (ICC 0.749 to 0.932). The total score and the physical domain showed similar variation, from moderate (lower limit) to strong (total score: 0.413 to 0.841 and physical domain: 0.434 to 0.847). This variability might be attributed to the CIEV's greater specificity towards the emotional domain and its relative lack of comprehensiveness compared to the DHI. Furthermore, the performance in the physical domain may influence the overall total score, contributing to the observed variation.

Finally, the correlation indices were validated using the Pearson’s correlation test to measure the extent to which the protocols are interconnected, based on the same theory. The CIEV findings in this analysis were statistically significant for both the total score and the DHI domains (p<0.001). In agreement with data discussed before, only the analysis of the total score and the emotional domain presented strong correlations (r=0820 and r=0.788, respectively).

Based on the results obtained in our study, the analyses of reliability and validation suggest the CIEV can be used as a complementary clinical instrument to identify the risk/ of anxiety in patients who experience vertigo and/or dizziness.

Our study has a few limitations related to the sample and the validated instrument. Sample limitations are related to the small sample size and sample heterogeneity in relation to dizziness complaints reported. The questionnaires were applied to a sample of individuals treated at a specific outpatient clinic for patients complaining about balance disorders/dizziness at a university hospital, mostly female (25; 65.75%) and elderly (20; 52.63%) patients, and vertigo was the mostly frequently reported type of dizziness. These characteristics are similar to epidemiological studies that indicate a higher predisposition of female individuals to otoneurological alterations associated with vertigo, especially due to metabolic and hormonal conditions^(23,24)^, more women seeking medical help^(25)^, and a higher prevalence of dizziness in elderly patients^(23)^. On the other hand, the sample was heterogeneous in relation to different comorbidities associated with dizziness and presented a higher occurrence of mixed hypothesis for the vestibular lesion topography, including vascular, metabolic, hormonal causes or a combination of peripheral and central causes. Additionally, some variables were not fully controlled, such as use of medication and previous dizziness treatments, and it was not possible to divide the participants into study groups according to the probable etiology of dizziness and/or specific disorders or vestibular lesion status.

Such sample heterogeneity agrees with a recent systematic review that analyzes the distribution of proportion of the sample diagnosed with dizziness/vertigo and the variations of this proportion over time^(25)^. The authors argue that, because dizziness is considered a symptom of different causes that may be related to several diseases^(26)^, a wide variation is observed in the diagnostic proportion, particularly depending on the specialties involved in the diagnosis and associated general health variables. Therefore, the study sample, despite being heterogeneous, can be considered representative of the diversity of cases that are admitted to the outpatient clinic based on the initial complaint of dizziness, which is often nonspecific.

Based on the data obtained in our study, the CIEV construct demonstrates strong internal consistency and acceptable reliability and validity indices, indicating its potential as an alternative tool for identifying emotional aspects related to vertigo. However, it is essential to note that the version used in our study was a freely translated version into Brazilian Portuguese by the authors of the instrument, lacking linguistic and cultural adaptation of the questions.

Finally, our findings provide valuable contributions to the validation of the CIEV and its potential integration into clinical practice. Given the initial validation data presented here, it is crucial to conduct further studies to validate the questionnaire with specific groups, explore its correlation with instrumented evaluations, and analyze its accuracy to discuss sensitivity and specificity as a predictor of pathological anxiety in various conditions. Additionally, longitudinal studies are needed to examine the questionnaire's application not only in the diagnostic process but also as a tool for therapeutic monitoring.

## CONCLUSION

Based on our study findings, the CIEV exhibited high internal consistency scores among its questions and demonstrated moderate to strong correlations with the DHI, particularly in the emotional domain. These findings were determined based on the mean results and the adopted confidence interval.. Hence, the findings of this study represent an important contribution to the validation of the CIEV questionnaire for clinical use in Brazil with a focus on identifying emotional aspects related to vestibular disorders complaints.
