# Supplementary material for: Validation and reliability analysis of the Questionnaire on the Emotional Impact of Vertigo-CIEV version translated to the Brazilian Portuguese language
Source: Codas. 2023 Dec 4;35(6):e20220176. doi: 10.1590/2317-1782/20232022176en (PMC10750823; doi:10.1590/2317-1782/20232022176en)
Supplement: Translation

# Translation: Análise de validação e confiabilidade do Questionário Emocional da Vertigem-CIEV em versão traduzida para o Português-Brasileiro

**DOI:** 10.1590/2317-1782/20232022176pt

**Authors:** Maria Luiza di Carlo Riato, Vanessa Brito Campoy Rocha, Raquel Mezzalira, Guita Stoler, Maria Isabel Ramos do Amaral

**Affiliations:** 1 Programa de Graduação em Fonoaudiologia, Faculdade de Ciências Médicas, Universidade Estadual de Campinas – UNICAMP - Campinas (SP), Brasil.; 2 Departamento de Desenvolvimento Humano e Reabilitação, Faculdade de Ciências Médicas, Universidade Estadual de Campinas – UNICAMP - Campinas (SP), Brasil.; 3 Departamento de Otorrinolaringologia, Cabeça e Pescoço, Faculdade de Ciências Médicas, Universidade Estadual de Campinas – UNICAMP - Campinas (SP), Brasil.

**Keywords:** Tontura, Vertigem, Avaliação de Sintomas, Estresse Emocional, Questionários

## Abstract

**Objetivo:**

Determinar a consistência interna e confiabilidade do “Questionário de Impacto Emocional da Vertigem-CIEV” e validar o instrumento em relação ao Dizziness Handicap Inventory (DHI), em uma amostra de pacientes com distúrbio do equilíbrio corporal.

**Método:**

Participaram 38 sujeitos, idades entre 23 e 85 anos, ambos os sexos, com queixas relacionadas à tontura, desequilíbrios e/ou quedas, atendidos em um ambulatório de Otoneurologia do Hospital Universitário. Foram excluídos sujeitos com queixas auditivas e/ou zumbido sem tontura associada, comorbidades psiquiátricas prévias e/ou comprometimento cognitivo que impedisse a compreensão dos questionários. Foi realizada anamnese, levantamento de prontuário para caracterização da amostra e aplicados os questionários de autopercepção, DHI e CIEV. O alfa de Cronbach verificou a consistência interna do CIEV e a confiabilidade e validade do CIEV em relação ao DHI foram calculadas pelo Índice de Correlação intraclasse (ICC) e teste de Correlação de Pearson, respectivamente.

**Resultados:**

Houve correlação estatisticamente significante entre os escores obtidos, tanto à análise de confiabilidade quanto de validação (p<0,001). O ICC médio demonstrou moderada correlação para o escore total (0,695) e forte correlação com os domínios físico, emocional e funcional do DHI (0,706 a 0,869) sendo o maior grau para o domínio emocional (0,869). A Correlação de Pearson demonstrou grau forte para o escore total (r=0,820) e variação de moderado a forte para os domínios, com melhor resultado também para o domínio emocional do DHI (r=0,788).

**Conclusão:**

Os achados representam parâmetros importantes de contribuição para a validação do CIEV para uso clínico na população brasileira, direcionado para a identificação de aspectos emocionais em pacientes com distúrbios do equilíbrio corporal.

## INTRODUÇÃO

O desequilíbrio e a tontura ocorrem na ausência ou falha da integração das informações provenientes dos sistemas visual, vestibular e/ou proprioceptivo, caracterizados por um processamento incorreto das aferências sensoriais, sendo que tais alterações podem estar relacionados a inúmeros fatores etiológicos^(1)^. A ocorrência dos sintomas acarreta prejuízos aos indivíduos, influencia a realização das atividades diárias e diminui a qualidade de vida^(2)^. Tal condição afeta principalmente os idosos, sendo que 15 a 20% da população mundial sofre ou já sofreu algum tipo de tontura^(3)^.

Nos últimos anos, o campo da neuro-otologia, que se dedica ao estudo dos distúrbios do equilíbrio corporal, tem apresentado significativos avanços clínico-científicos. Os consensos e diretrizes clínicas atuais possibilitam um direcionamento importante para o correto diagnóstico dos diferentes quadros que acometem o sistema vestibular, aliados a avaliação física e instrumentada e exames de imagem cada vez mais precisos. Mas, mesmo diante de tantos avanços, a história clínica e o auto relato do paciente permanecem como sendo o ponto fundamental tanto para a etapa diagnóstica quanto para o adequado planejamento e monitoramento terapêutico do paciente com distúrbios vestibulares^(4)^.

Dentre os muitos fatores que podem estar associados a ocorrência da tontura e que devem ser investigados, destacam-se nesse estudo os aspectos emocionais. Nas últimas décadas, evidenciou-se a relação entre as questões psicológicas e a vertigem^(5,6)^. A existência da relação de causa e efeito ou ainda da concomitância dos sintomas vêm sendo investigada devido às vias e conexões em comum entre o sistema vestibular e o sistema límbico^(5)^. Estudos apontam que sujeitos com queixas otoneurológicas mostram-se mais susceptíveis a apresentar problemas psicológicos e um estilo de vida mais estressante do que pacientes sem queixa, sendo que os indivíduos sem queixas possivelmente apresentam melhores mecanismos fisiológicos para lidar com o estresse^(7)^.

A compreensão e a identificação de sintomas emocionais e otoneurológicos, bem como o impacto de um sintoma sob o outro pode garantir uma melhor condução do caso clínico^(5)^. Essa compreensão não é uma tarefa fácil e, muitas vezes, apenas a avaliação dos sintomas pode não ser suficiente. Mesmo em períodos assintomáticos, a tontura pode comprometer diferentes aspectos da vida diária devido a ansiedade e medo da antecipação de um próximo episódio, mais do que a ocorrência do sintoma em si^(8)^. Sendo assim, entende-se que a etapa diagnóstica pode conter lacunas na identificação de problemas psicológicos associados ao quadro da tontura, sendo uma relação de difícil reconhecimento na primeira avaliação do paciente vertiginoso, uma vez que a ansiedade pode ou não estar presente no momento inicial^(9)^.

Os questionários de autopercepção têm sido recomendados como instrumentos que permitem com que sintomas amplos, subjetivos e de difícil caracterização, tais como a tontura e seus diferentes impactos na qualidade de vida, sejam quantificados e analisados^(10)^. O protocolo *Dizziness Handicap Inventory* (DHI), proposto por Jacobson e Newman^(11)^ consolidou-se como o instrumento mais utilizado em pesquisas e na prática clínica para este fim, validado em diferentes países, incluindo o Brasil^(12)^ e ainda amplamente estudado. Recentemente, uma versão reduzida foi proposta, sendo defendida pelos autores como uma alternativa facilitadora em relação à versão original, para acessar tais aspectos durante a prática clínica^(13)^.

Como proposta de mais um instrumento clínico dessa natureza, o “Questionário de Impacto Emocional da Vertigem – CIEV” foi elaborado e validado em língua espanhola^(14)^ com o objetivo de mensurar o risco para a ocorrência de quadros de ansiedade patológica secundários à vertigem, a partir da autopercepção dos sintomas, como uma estratégia complementar à avaliação diagnóstica. No Brasil, o CIEV teve sua versão original traduzida livremente pelos autores e publicada em Português^(15)^. No entanto, até o momento, não foram encontrados estudos nacionais de validação deste potencial instrumento, sendo importante estudar e discutir suas propriedades clinimétricas e validade do constructo.

Diante do exposto, o objetivo desta pesquisa foi determinar a consistência interna e confiabilidade do “Questionário de Impacto Emocional da Vertigem-CIEV” e validar o instrumento em relação ao *Dizziness Handicap Inventory* (DHI), em uma amostra de pacientes com distúrbio do equilíbrio corporal sem diagnóstico prévio de alterações psiquiátricas atendidos em um Hospital Universitário.

## MÉTODO

### Tipo, local do estudo e aspectos éticos

Trata-se de um estudo de confiabilidade e validade, quantitativo, descritivo e de corte transversal. A coleta de dados ocorreu no Ambulatório de Otoneurologia do Hospital Universitário da Instituição, após a aprovação do Comitê de Ética em Pesquisa (#2.344.836). Todos os sujeitos consentiram com a participação voluntária e assinaram o Termo de Consentimento Livre e Esclarecido (TCLE).

### Participantes e critérios de seleção

A partir da rotina do Ambulatório, os participantes foram selecionados e avaliados no dia da primeira consulta de avaliação. A pesquisadora signatária deste estudo acompanhou os atendimentos junto à equipe médica e, ao final da consulta, aqueles que preenchiam os critérios de inclusão foram convidados a participar.

Os critérios de inclusão foram: sujeitos de ambos os sexos com idades a partir de 18 anos e que apresentassem queixas relacionadas à tontura/vertigem e eventualmente, histórico de desequilíbrios e/ou quedas. Foram excluídos sujeitos que tinham algum grau de comprometimento cognitivo de forma que impedisse a compreensão e realização dos questionários, bem como sujeitos que apresentassem exclusivamente uma queixa auditiva e/ou zumbido, sem a ocorrência da tontura. Também foram excluídos os sujeitos que referiram comorbidades psiquiátricas prévias no momento da anamnese e/ou registro em prontuário médico.

### Caracterização da amostra

Os dados referentes a caracterização da amostra foram obtidos a partir dos seguintes procedimentos:

- Anamnese Otoneurológica: aspectos associados à queixa (tipo da tontura e tempo da queixa), histórico de quedas pregressas nos últimos 12 meses referente a data da avaliação, sintomas auditivos associados (perda auditiva e/ou zumbido), medicamentos utilizados e saúde geral.
- Levantamento de prontuário médico: nesta etapa, os dados referentes a anamnese foram confirmados, foi feito o levantamento das hipóteses da topografia da lesão vestibular atribuída a cada caso e presença de comorbidades. Quando necessário, foram realizadas discussões com a equipe médica para confirmação das informações.

O tipo de tontura foi classificado de acordo com a Classificação Internacional das Desordens Vestibulares^(16)^, sendo: tontura não rotatória, vertigem, sintomas posturais e sintomas vestíbulo-visuais. Os dados referentes à hipótese da topografia da lesão de cada sujeito, coletados a partir de levantamento do prontuário após a avaliação médica, foram agrupados entre periférica, central, mista, cervical ou “a esclarecer”. A categoria mista incluiu doenças de etiologia vascular, metabólicas, hormonais e ainda quando periféricas e centrais combinadas. Além disso, na categoria “a esclarecer” foram incluídas etiologias proprioceptivas, exames otoneurológicos inconclusivos e/ou normais.

A amostra foi composta por 38 sujeitos, com idades entre 23 e 85 anos, média de 58,66 anos (±16,37), sendo 25 (66,79%) mulheres. O tempo de queixa variou entre 1 semana e 4,87 anos. Considerando os últimos 12 meses da data em que o sujeito foi avaliado, 16 (38%) participantes referiram queda pregressa relacionada com a tontura. 23 (60,53%) sujeitos apresentavam algum tipo de perda auditiva e 26 (68,42%) referiram zumbido. A Tabela 1 apresenta a caracterização da amostra estudada com relação ao tipo de tontura referido, comorbidades e distribuição da hipótese topográfica da lesão vestibular. Alguns sujeitos referiram mais de um tipo de queixa e mais de uma comorbidade. Além disso, a hipótese cervical foi identificada como hipótese topográfica única em apenas dois participantes, em outros seis casos foram levantados mais de uma hipótese associada com a questão cervical.

**Tabela 1:** Distribuição absoluta e frequência relativa da classificação do tipo de queixa apresentada, topografia da lesão vestibular e comorbidades identificadas em cada caso, considerando o levantamento de dados obtidos junto ao prontuário médico

**Tipo de tontura**	**n**	**%**	**Distribuição Topográfica**	**n**	**%**	**Comorbidades**	**n**	**%**
Vertigem	24	48,97	Mista	22	50,00	Endocrinológicas	16	25,80
Sintomas posturais	17	34,70	Cervical	8	18,18	Cardiovasculares	15	24,20
Tontura não-rotatória	7	14,28	Central	7	15,90	Reumatológica	8	12,90
Sintoma vestíbulo-visual	1	2,04	A esclarecer	4	9,10	Neurológicas	7	11,30
-	-	-	Periférico	3	6,81	Hematológicas	5	8,06
-	-	-	-	-	-	Respiratórias	4	6,45
-	-	-	-	-	-	Autoimunes	3	4,84
-	-	-	-	-	-	Obesidade	2	3,23
-	-	-	-	-	-	Linfáticas	1	1,61
-	-	-	-	-	-	Oncológicas	1	1,61
**Total**	**49**	**100,0**	**Total**	**44**	**100,0**	**Total**	**62**	**100,0**

Legenda: n = frequência absoluta

### Coleta de dados

Anteriormente à etapa de coleta de dados, foi realizado um estudo piloto com o objetivo de treinar e padronizar a aplicação dos questionários de pesquisa. Inicialmente, a pesquisadora explicou o questionário ao participante e a leitura das perguntas foi feita juntamente com o participante. Após a resposta, a pesquisadora marcou a opção escolhida na folha do protocolo. Os dois questionários aplicados serão descritos a seguir:

- *Dizziness Handicap Inventory* (DHI)^(12)^ – formado por 25 questões, as quais avaliam três domínios relacionados ao possível impacto/prejuízo na qualidade de vida ocasionado pela tontura. São sete questões que avaliam os aspectos físicos, nove relacionadas aos aspectos emocionais e sete aos funcionais. As possíveis respostas são “sim” (4 pontos), “às vezes” (2 pontos) e “não” (zero pontos). O escore final é dado pela somatória de todos os valores, variando de zero a 100. Além disso, também foram tabulados os escores por domínio físico, funcional e emocional. A classificação quanto ao nível de impacto ocasionado pela tontura é baseada no escore total e dividida por graus de prejuízo, portanto, de 0 a 30 pontos o sujeito recebe classificação de baixo impacto emocional, 31 a 60 pontos, médio impacto e 61 a 100 pontos, alto impacto.
- Questionário de Impacto Emocional da Vertigem (CIEV)^(15)^– dividido em três partes, que totalizam 19 perguntas. A parte I é *introdutória* a respeito da definição de tontura e vertigem relatada pelo paciente e a pergunta não tem pontuação atribuída. As demais 18 perguntas estão distribuídas na partes II- *Experiência durante os episódios de vertigem/tontura* e parte III - *situações vivenciadas relacionadas ao grau de angústia associado*. Em cada uma das etapas II e III, às três opções de resposta atribui-se as pontuações 0, 1 ou 2, sendo que a pontuação zero é relacionada com a resposta “Nunca” ou “Não, isso nunca me aconteceu”, um ponto é atribuído às respostas “às vezes” ou “Sim, e isso me deixa um pouco angustiado” e dois pontos é o valor atribuído as respostas “muitas vezes” e “Sim, isso me angustia muito”. Portanto, o escore final varia de 0 a 36 pontos. Resultados acima de 16 pontos são classificados como risco para ansiedade patológica e/ou risco para um pior prognóstico no tratamento da tontura^(14,15)^. O questionário CIEV pode ser visualizado na íntegra no Quadro 1.

**Quadro 1:** Questionário do Impacto Emocional da Vertigem – CIEV

**Questionário de impacto emocional da vertigem – CIEV**

Dados pessoais:
Nome completo: ______________________________________ DN: __________ idade: ________
Profissional responsável: __________________________________________________________

**I) Introdução:**
Entendemos por vertigem como a sensação de rotação do próprio corpo ou do entorno geralmente é um movimento rotatório). Exemplo: “As coisas giram...” Por outro lado, a tontura refere-se a uma sensação de desequilíbrio. Exemplo: “Andar nas nuvens...”. Qual dessas duas definições coincide mais com o que você sente?
() Vertigem () Tontura

**I) Responda de acordo com a sua experiência durante os episódios de vertigem/tontura:**

1. Enquanto estava com vertigem/tontura sentiu que estava perdendo controle sobre seu corpo?
NUNCA () AS VEZES () MUITAS VEZES ()

2. Enquanto estava com vertigem/tontura pensou que poderia desmaiar ou desfalecer?
NUNCA () AS VEZES () MUITAS VEZES ()

3. Enquanto estava com vertigem/tontura se sentiu desprotegido, sem ninguém para te socorrer? |
NUNCA () AS VEZES () MUITAS VEZES ()

4. Enquanto estava com vertigem/tontura sentiu ansiedade e medo de ser “dominado”?
NUNCA () AS VEZES () MUITAS VEZES ()

5. Enquanto estava com vertigem/tontura teve sintomas como taquicardia, sudorese ou asfixia?
NUNCA () AS VEZES () MUITAS VEZES ()

6. Enquanto estava com vertigem/tontura e ao sentir que havia muitas pessoas no local, seu mal-estar aumentava?
NUNCA () AS VEZES () MUITAS VEZES ()

7. Enquanto estava com vertigem/tontura sentiu que estava melhor na sua casa do que fora dela?
NUNCA () AS VEZES () MUITAS VEZES ()

8. Enquanto estava com vertigem/tontura sentia-se melhor se alguém de confiança estivesse
perto de você?
NUNCA () AS VEZES () MUITAS VEZES ()

|-----------------------------------------------------------------------------------------------|
Com a presença da sintomatologia de vertigem/tontura, muitas pessoas experimentam mudanças na forma de ser ou agir que as fazem sentir-se realmente “diferentes” de como sempre foram. A seguir é detalhada uma série de experiências frequentes de pacientes com problemas semelhantes aos seus.

**II) Responda se isso já aconteceu com você e, se a resposta for positiva, o quanto você se sente angustiado ao perceber essa mudança na sua maneira de ser.**

**1. Sente estar dependendo mais da ajuda ou da companhia de outras pessoas?**
A) Não, isso nunca me aconteceu. ()
B) Sim, e isso me deixa um pouco angustiado. ()
C) Sim, e isso me angustia muito. ()

**2. Sente que está mais sensível? (Ex.: Chora com mais facilidade,...)**
A) Não, isso nunca me aconteceu. ()
B) Sim, e isso me deixa um pouco angustiado. ()
C) Sim, e isso me angustia muito. ()

**3. Sente estar mais irritado, menos tolerante com as pessoas?**
A) Não, isso nunca me aconteceu. ()
B) Sim, e isso me deixa um pouco angustiado. ()
C) Sim, e isso me angustia muito. ()

**4. Sente que está mais medroso, que se assusta com mais facilidade do que antes?**
A) Não, isso nunca me aconteceu. ()
B) Sim, e isso me deixa um pouco angustiado. ()
C) Sim, e isso me angustia muito. ()

**5. Você pensa na possibilidade de sofrer doenças graves?**
A) Não, isso nunca me aconteceu. ()
B) Sim, e isso me deixa um pouco angustiado. ()
C) Sim, e isso me angustia muito. ()

**6. Sente temor de ficar sozinho em casa ou viajar sem companhia?**
A) Não, isso nunca me aconteceu. ()
B) Sim, e isso me deixa um pouco angustiado. ()
C) Sim, e isso me angustia muito. ()

**7. Você costuma evitar os lugares cheios de gente? (supermercados, cinemas, shoppings, etc)**
A) Não, isso nunca me aconteceu. ()
B) Sim, e isso me deixa um pouco angustiado. ()
C) Sim, e isso me angustia muito. ()

**8. Você acha que está suportando menos as “pressões” profissionais e familiares?**
A) Não, isso nunca me aconteceu. ()
B) Sim, e isso me deixa um pouco angustiado. ()
C) Sim, e isso me angustia muito. ()

**9. Você sente que fica difícil “lidar” com situações simples, que antes você controlava melhor?**
A) Não, isso nunca me aconteceu. ()
B) Sim, e isso me deixa um pouco angustiado. ()
C) Sim, e isso me angustia muito. ()

**10. Você se sente menos forte, com menos “capacidade para lutar” do que antes?**
A) Não, isso nunca me aconteceu. ()
B) Sim, e isso me deixa um pouco angustiado. ()
C) Sim, e isso me angustia muito. ()

**Escore: _________**

“Nunca” e “Não, isso nunca me aconteceu” = zero pontos
“Às vezes” e “Sim, e isso me deixa um pouco angustiado” = um ponto
“Muitas vezes” e “Sim, e isso me angustia muito” = dois pontos
|-----------------------------------------------------------------------------------------------|

### Análise estatística

Os dados foram analisados por meio da estatística descritiva e inferencial, utilizando-se os *softwares* SPSS 25.0, *Minitab* 16 e *Excel Office* 2010. A normalidade das variáveis quantitativas de desfecho principal foi confirmada a partir do teste de *Shapiro Wilks* e adotou-se um nível de significância de 5% para todas as análises inferenciais. Os valores estatisticamente significantes (p<0,05) foram destacados em negrito. Para a descrição das variáveis quantitativas foram calculadas a medida de tendência central (média), a medida de variabilidade (desvio-padrão) e as medidas de posição (mínimo e máximo). Para a descrição das variáveis qualitativas nominais foram calculadas a frequência absoluta e a frequência relativa percentual.

O coeficiente *alfa de Cronbach* foi calculado para análise da consistência interna dos dados do questionário CIEV, a partir da medida de correlação média entre as questões. Esse coeficiente tem seu máximo valor em 1,0 e quanto maior é o valor, maior é a consistência interna dos dados analisados. Valores de alfa acima de 0,7 são considerados aceitáveis. Essa análise foi realizada considerando a pontuação total do CIEV e também a partir da retirada a cada momento de uma das questões.

O cálculo de Índice de Correlação intraclasse (ICC) foi realizado para verificar a confiabilidade do protocolo estudado e a validade foi verificada a partir do teste de Correlação de *Pearson*, ambas análises por meio da comparação com o escore total do CIEV com o escore total e por domínios obtidos no protocolo DHI. Os valores de ICC e correlação variam de 0 a 1, sendo que valores mais próximos de 1 indicam melhor grau de correlação. No cálculo do ICC, além do valor médio, foi apresentado o ICC baseado no limite inferior e superior do intervalo de confiança adotado.

Para os coeficientes de correlação (r) e ICC foi assumido: |0,10| a |0,39| - correlação fraca; |0,40| a |0,69| - correlação moderada; |0,7| a |1,00| - correlação forte^(17)^.

## RESULTADOS

O desempenho da amostra estudada no CIEV, considerando o escore final e classificação de risco para a ansiedade patológica, podem ser visualizados na Tabela 2. E a Tabela 3 apresenta o desempenho da amostra no questionário DHI, considerando o escore total, escore em cada domínio e classificação do grau de impacto da tontura (*handicap).*

**Tabela 2:** Descrição da pontuação obtida no questionário CIEV e a classificação de risco para ansiedade patológica (n=38)

**Questionário de Impacto Emocional da Vertigem (CIEV)**						
Pontuação	n	Min	Max	Média	Mediana	Desvio Padrão
	38	5,0	35,0	20,39	20,00	8,48
**Classificação**	**n**			**%**		
**Não risco**	8			21,05%		
**Risco**	30			78,95%		

Análise descritiva

Legenda: n = frequência absoluta; % = frequência relativa percentual; Min = mínimo; Max = máximo

**Tabela 3:** Descrição do escore obtido no questionário DHI, bem como a classificação do impacto do *handicap* percebido, considerando a classificação total e os domínios funcional, físico e emocional (n=38)

**DIZZINESS HANDICAP INVENTORY (DHI)**								
	**Escore**							
	**Min**		**Max**		**Média**		**Desvio Padrão**	
**Funcional**	0		36		17,95		9,05 7,81 10,60 23,00	
**Físico**	0		28		16,90			
**Emocional**	0		36		14,62			
**Total**	16		92		51,47			
**Classificação (*Handicap*)**	**Baixo**		**Moderado**		**Alto**		**Total**	
	**n**	**%**	**n**	**%**	**n**	**%**	**n**	**%**
	9	23,68	15	39,47	14	36,84	38	100

Análise descritiva

Legenda: n = frequência absoluta; % = frequência relativa percentual; Min = mínimo; Max = máximo

A Figura 1 demonstra a análise de consistência interna do questionário CIEV. O alto valor de alfa obtido para a pontuação total (0,858) se manteve estável, com variação de 0,841 a 0,893 a partir da análise com base na retirada de cada questão.

**Figura 1:** Consistência interna do protocolo CIEV por meio da análise de Alfa de Cronbach (n = 38)

Os dados de confiabilidade e de validade do protocolo CIEV em relação ao escore total e por domínios do DHI podem ser visualizados na Tabela 4. Os achados foram estatisticamente significantes (p<0,001), sendo que os valores médios relacionados ao escore total demonstraram moderada correlação quanto a confiabilidade (0,695) e forte correlação quanto a validade do instrumento, também com base no escore total (0,820).

**Tabela 4:** Análise da confiabilidade e validação do protocolo CIEV em relação ao escore total e domínios avaliados pelo protocolo DHI

**DHI**	**Confiabilidade - ICC**				**Validação**	
	**ICC**	**P-valor**	**Lim. Inferior**	**Lim. Superior**	**R**	**P-valor**
**Escore Total**	0,695	**<0,001**	0,413	0,841	0,820	**<0,001**
**Domínios**						
**Físico**	0,706	**<0,001**	0,434	0,847	0,547	**<0,001**
**Emocional**	0,869	**<0,001**	0,749	0,932	0,788	**<0,001**
**Funcional**	0,809	**<0,001**	0,632	0,901	0,681	**<0,001**

Legenda: ICC = Índice correlação intraclasse; Lim. = Limite; R = Coeficiente de Correlação/Teste de Correlação de Pearson

## DISCUSSÃO

Instrumentos de medida, especialmente aqueles que se propõem a mensurar habilidades ou domínios a partir de vários itens que resultam em um escore final, tal como o CIEV, podem ser considerados úteis clinicamente a partir do momento que têm suas propriedades clinimétricas conhecidas, ou seja, dados de confiabilidade, validade e acurácia bem estabelecidos^(18)^. Na presente amostra, o CIEV foi analisado com base no estudo da confiabilidade e validade de seu constructo, a partir das medidas de consistência interna e dos graus de correlação com o DHI, um questionário derivado da teoria semelhante.

O DHI foi selecionado por ser um instrumento validado e estabelecido para uso clínico. Pesquisas demonstram ser um questionário confiável, abrangente, de fácil aplicação e adequado para a avaliação clínica de pacientes acometidos pela tontura, a partir da mensuração do grau de impacto sob a qualidade de vida que possa estar relacionado especificamente com a desordem vestibular^(12,19)^. Ainda assim, mesmo amplamente utilizado e pesquisado, a literatura aponta lacunas e perspectivas de mais estudos tanto com o DHI como outros questionários semelhantes, o que justifica a elaboração e validação de novos instrumentos. Dentre essas lacunas, destacam-se poucos estudos voltados para a investigação da relação entre os escores e doenças vestibulares específicas ou status clínico da lesão vestibular e a relação com métodos objetivos de avaliação da função vestibular^(20)^.

Quanto aos resultados da presente pesquisa em relação ao desempenho em cada um dos questionários aplicados, 30 sujeitos (78,95%) foram classificados como risco de desenvolver ansiedade patológica pelo CIEV e 29 (76,31%) apresentaram escores alterados no DHI. Destaca-se ainda que, dos 30 sujeitos com escores alterados no CIEV, 25 (83,3%) também foram classificados como médio e alto impacto no DHI. O desempenho da amostra corrobora com os estudos que reforçam a associação entre os aspectos emocionais e a tontura^(5-9)^, especialmente a ansiedade^(21)^.

O desempenho descrito condiz também com o estudo original de elaboração e validação do CIEV^(14)^. Os autores tiveram como objetivo avaliar o quadro emocional de pacientes com tontura persistente mesmo após tratamento, a partir da aplicação do CIEV. Dos 183 sujeitos avaliados, 157 (86%) apresentavam histórico confirmado de ansiedade patológica e destes, 60 (69,7%) tiveram resultados alterados no CIEV. Apesar das diferenças metodológicas na característica da amostra selecionada em relação ao presente estudo, a pesquisa demonstrou a associação entre os sintomas e também reforçou a necessidade de estudos de validação para uso complementar do CIEV na prática clínica.

A consistência interna representa um indicador de confiabilidade de medida e estabilidade do instrumento, demonstrada a partir da análise de correlação média entre as respostas dos participantes^(22)^. Os dados obtidos demonstraram alta consistência interna entre as questões do protocolo e escore total. Na verificação dos dados a partir da retirada a cada momento de uma das questões, a estabilidade pôde ser confirmada, uma vez que os valores de alfa se mantiveram altos. O menor valor obtido foi referente a questão 4, relacionada a sensação de medo após o início da tontura, mas ainda assim, alto (0,841).

Na análise do ICC, foram encontrados valores estatisticamente significantes (p<0,001) em relação tanto ao escore total do DHI (r=0,695) quanto as três dimensões avaliadas (física, funcional e emocional), com destaque para o domínio emocional, com o maior grau médio de correlação positiva classificado como forte (r=0,869). Ao considerarmos a variação quanto aos valores de limite inferior e superior baseados no intervalo de confiança de 95% adotado, o domínio emocional apresentou menor variação, demonstrando uma correlação forte (ICC 0,749 a 0,932). Já o escore total e o domínio físico apresentaram variação semelhante, de moderada (limite inferior) a forte (escore total: 0,413 a 0,841 e domínio físico: 0,434 a 0,847). Uma justificativa que pode ser discutida em relação a essa variação pode estar relacionada com o fato do CIEV se apresentar como um questionário mais específico do domínio emocional e menos abrangente que o DHI e a possível influência do desempenho no domínio físico sob o escore total.

Por fim, os índices de correlação foram validados através do Teste de Correlação de Pearson, aplicado a fim de mensurar o quanto os protocolos encontram-se interligados, a partir de uma mesma teoria. Os achados do CIEV em relação a esse instrumento na análise citada foram estatisticamente significantes tanto para o escore total quanto domínios do DHI (p<0,001). Corroborando com os dados discutidos anteriormente, apenas a análise do escore total e do domínio emocional apresentaram correlações consideradas fortes (r=0,820 e r=0,788, respectivamente).

Com base nos resultados discutidos, é possível afirmar que as análises de confiabilidade e validação confirmaram a possibilidade do uso do CIEV como um instrumento clínico complementar voltado para a identificação do risco/tendência a quadros ansiosos em pacientes que experienciam vertigem e/ou tontura.

O estudo apresenta limitações a serem discutidas, relacionadas a amostra e também ao instrumento validado. As limitações da amostra não estão relacionadas apenas ao tamanho amostral reduzido, mas também em relação a heterogeneidade da amostra em relação ao quadro de tontura. Os questionários foram aplicados em uma amostra de indivíduos atendidos em ambulatório específico direcionado para pacientes com queixas relacionadas as desordens do equilíbrio/tontura de um Hospital Universitário, sendo composta em sua maioria por mulheres (25/65,75%) e idosos (20/52,63%) e a vertigem foi o tipo de tontura mais referido. Essas características se assemelham a estudos epidemiológicos que apontam maior predisposição do sexo feminino em sofrer alterações otoneurológicas associadas a vertigem, especialmente devido a quadros metabólicos e hormonais^(23,24)^, mais busca de ajuda médica pelas mulheres^(25)^, assim como maior prevalência da tontura em idosos^(23)^. Por outro lado, a amostra foi heterogênea em relação as diferentes comorbidades associadas ao quadro da tontura e maior ocorrência da hipótese mista para a topografia da lesão vestibular, incluindo causas de origem vascular, metabólicas, hormonais ou combinadas entre causas periféricas e centrais. Sendo assim, algumas variáveis não foram controladas ou totalmente controladas como o uso de medicação, tratamentos prévios já realizados para a tontura, e não foi possível a subdivisão em grupos de estudo classificados segundo a provável etiologia da tontura e/ou doenças específicas ou até mesmo status da lesão vestibular.

Essa heterogeneidade na amostra corrobora com a discussão apresentada em revisão sistemática recente a respeito da proporção da distribuição de diagnósticos para pacientes com tontura/vertigem e as variações dessa proporção ao longo do tempo^(25)^. Os autores discutem que pelo fato de a tontura ser considerada um sintoma de origem multifatorial que pode estar relacionado a inúmeras doenças^(26)^, há uma grande variação nessa proporção diagnóstica, principalmente a depender das especialidades envolvidas no diagnóstico e variáveis de saúde associadas. Portanto, a amostra estudada, apesar de heterogênea, pode ser considerada representativa da diversidade de casos que dão entrada no Ambulatório a partir da queixa inicial de tontura, por vezes, inespecífica.

Os dados obtidos evidenciaram que o construto CIEV demonstra ser um protocolo com alta consistência interna e aceitáveis índices de confiabilidade e validade, tornando-o uma alternativa em potencial para a identificação de aspectos emocionais relacionados a vertigem, ainda que tenham sido obtidos a partir de uma versão traduzida livremente para o Português brasileiro pelos autores do instrumento, sem que fosse realizada a etapa de adaptação linguística e cultural das questões.

Portanto, os resultados alcançados contribuem para a validação do CIEV a ser inserido na prática clínica e, com base nos dados iniciais de validação aqui apresentados, mais estudos devem ser realizados, direcionados tanto para a validação em grupos específicos de pacientes, associação com as avaliações instrumentadas, análise de acurácia para discussão dos dados de sensibilidade e especificidade do instrumento como preditivo da ansiedade patológica nos diferentes quadros, bem como estudos de caráter longitudinal que possam discutir a aplicação do questionário não somente durante a etapa diagnóstica, mas também como instrumento de monitoramento terapêutico.

## CONCLUSÃO

Considerando a amostra estudada e os resultados apresentados, o CIEV demonstrou escores com alta consistência interna entre suas questões, e correlações com o DHI que variaram de moderada a fortes, considerando os resultados médios e intervalo de confiança adotado, com destaque para o domínio emocional. Sendo assim, os achados desse estudo representam parâmetros importantes de contribuição para a validação do questionário CIEV para uso clínico no Brasil, direcionado para a identificação de aspectos emocionais relacionados a queixas otoneurológicas.
